# Pyrolysis Kinetics Analysis and Prediction for Carbon Fiber-Reinforced Epoxy Composites

**DOI:** 10.3390/polym15234533

**Published:** 2023-11-25

**Authors:** Pei Xiao, Jingyi Zhang, Han Li, Haolei Mou, Zhenyu Feng, Jiang Xie

**Affiliations:** College of Safety Science and Engineering, Civil Aviation University of China, Tianjin 300300, China; dbxtxp@163.com (P.X.); zhangjy24@foxmail.com (J.Z.); cauc_lihan@126.com (H.L.); mhl589@163.com (H.M.); mhfzy@163.com (Z.F.)

**Keywords:** pyrolysis kinetics analysis, carbon fiber-reinforced epoxy composites, pyrolysis prediction model

## Abstract

Carbon fiber-reinforced epoxy resin composites have poor high temperature resistance and are prone to thermal damage during service in the aerospace field. The purpose of this study was to evaluate the thermal decomposition (pyrolysis) characteristics of carbon fiber-reinforced epoxy composites and reasonably predict their thermal decomposition under arbitrary temperature conditions. The kinetic analysis was conducted on the thermal decomposition of carbon fiber-reinforced epoxy resin composites (USN15000/9A16/RC33, supplied by Weihai GuangWei Composites Co., Ltd. Weihai City, Shandong Province, China) under a nitrogen environment, and an improved model of pyrolysis prediction suitable for the arbitrary temperature program was developed in this work. The results showed that the carbon fiber-reinforced epoxy composites begin to degrade at about 500 K, and the peak value of the weight loss rate at the respective heating rate appears in the range of 650 K to 750 K. A single-step reaction can characterize the thermal decomposition of carbon fiber-reinforced epoxy composites in a nitrogen atmosphere, and a wide variety of isoconversional approaches can be used for the calculation of the kinetic parameters. The proposed model of pyrolysis prediction can avoid numerous limitations of temperature integration, and it shows good prediction accuracy by reducing the temperature rise between sampling points. This study provides a reference for the kinetic analysis and pyrolysis prediction of carbon fiber-reinforced epoxy composites.

## 1. Introduction

Fiber-reinforced polymer composites have been extensively employed in the aerospace and aeronautics field in recent decades because of their excellent mechanical properties. Although aircraft made of composite materials are economical and environmentally friendly, the significant differences in physical and chemical properties between fiber reinforced composites and metal materials also present new problems for the safety of composite aircraft. Carbon fiber-reinforced polymer (CFRP) is likely to have thermal damage. The resin matrix of CFRP is flammable, and it releases toxic fumes as it burns [1]. Moreover, the electrical conductivity of CFRP currently used on aircraft is approximately 1000 times less than that of standard aluminum materials, while the electrical resistance of the composite resins is 1,000,000 times higher and usually considered to be insulating [2]. Some research has suggested that due to strong current, the composite structure will be ablated and fail [3,4,5,6].

The above problems are significantly correlated with the thermal decomposition (pyrolysis) characteristics of CFRP at high temperatures, whether it is burning or due to a lightning strike. Thermogravimetric analysis (TGA) has been used the most for the characterization of the thermal decomposition characteristics of materials. Some research has been conducted on the pyrolysis characteristics of CFRP matrix using the TGA method [7,8,9,10,11,12,13]. The kinetic parameters are obtained through the analysis of thermogravimetric data, and the mathematical relationship between conversion rate and temperature, also known as the conversion rate equation, is developed. The conversion rate equation has numerous applications, one of which is temperature response analysis of structures subjected to fire. Gibson et al. [14] slightly modified the thermal equation by Henderson et al. [15] to consider the conversion rate of the polymer matrix. Some studies on the thermal response of composites were derived [16,17,18,19,20,21,22], in accordance with the thermal response model established by Henderson et al. [15] and Gibson et al. [14].

Another critical application of kinetic analysis is kinetic prediction [23], which has been commonly adopted to evaluate the pyrolysis behavior of materials exceeding the temperature procedure of experimental conditions [24]. However, the pyrolysis of materials is closely related to the thermal damage of materials, and the development of the corresponding relationship between material thermal decomposition and thermal damage is of high significance in engineering fields (e.g., fire, explosion, and lightning). The thermal damage modeling of material mechanical properties has been primarily based on the temperature in composites exposed to fire [21,25,26,27,28] and lightning [29,30]. At temperatures lower than 200 °C, the compression failure of a composite is largely due to the glass transformation of the matrix. At higher temperatures, the failure of a composite is also dependent on the pyrolysis and delamination cracking of the matrix [25]. As revealed by the TGA data, the starting and ending temperatures of thermal decomposition are significantly related to the heating rates, and using only the temperature criterion cannot effectively characterize the thermal damage of the composite material. The time effect of temperature is considered in the pyrolysis degree, and using it is beneficial to characterize the relationship between decomposition and thermal damage of the material. However, the problem of temperature integration is inevitable when the rate equation is adopted to calculate the pyrolysis degree. The temperature integral cannot be integrated in a closed form, so rough and inaccurate approximations are employed in numerous kinetic analyses [31,32]. In addition, the temperature integral is only applicable to the linear heating condition with constant heating rate, and has nothing to do with the nonlinear heating condition widely common in engineering problems. Bai et al. [33] proposed a finite difference method to obtain the pyrolysis degree. However, the equation has a complex solution, and the accuracy should be verified. Dong et al. [34,35,36,37] and Kamiyama et al. [38] re-deduced the expression of pyrolysis degree under nonlinear heating conditions based on the rate equation for avoiding the limitation of the temperature integral, so as to obtain the change in conductivity with pyrolysis degree in the thickness direction of composite materials in the simulation analysis of lightning strike damage. However, the derivation is not rigorous since the isothermal assumption is unconsciously introduced in the derivation process. Accordingly, the main research topic of this study places a focus on the thermal decomposition prediction based on an arbitrary temperature program.

This study primarily aimed to obtain the pyrolysis kinetics parameters of carbon fiber-reinforced epoxy composites and develop a pyrolysis prediction model suitable for an arbitrary temperature program. In this study, the prediction model of pyrolysis under an arbitrary temperature program was deduced first. Second, the thermogravimetric (TG) test of CFRP composites was performed. Subsequently, several isoconversional methods were used for kinetic analysis, and the applicability of various isoconversional methods was compared. Lastly, the accuracy of the model of pyrolysis prediction was verified. This study provides a reference for the kinetic analysis and pyrolysis prediction of carbon fiber-reinforced epoxy composites.

## 2. Theory

### 2.1. Kinetic Analysis

The most extensively used kinetic analysis model on a single-step process considers the reaction rate as a function of temperature T and extent of conversion (pyrolysis degree) α [23]:

(1) $$\frac{d\alpha }{dt}=k\left(T\right)f\left(\alpha \right)$$

where the rate constant $k\left(T\right)$ denotes the dependence of the process rate on temperature; $f\left(\alpha \right)$ is the dependence on the extent of conversion, called the reaction model.

Here, the rate constant $k\left(T\right)$ is expressed by the Arrhenius equation involving the gas constant R, the activation energy E, and the pre-exponential factor A:

(2) $$k\left(T\right)=A\exp\left(\frac{-E}{RT}\right)$$

The conversion dependence of reaction rates can be expressed by a variety of reaction models, $f\left(\alpha \right)$. Table 1 lists some of the models. Notably, most of listed models are aimed at solid reactions [39].

Pyrolysis kinetic analysis aims to develop the mathematical relationship of conversion rate and temperature by solving the kinetic triplet, a term frequently adopted to express a set of A, E, $f\left(\alpha \right)$ [23].

At a constant heating rate, Equation (1) is written as below:

(3) $$\beta \frac{d\alpha }{dT}=A\exp\left(\frac{-E}{RT}\right)f\left(\alpha \right)$$

Integration of Equation (3) leads to:

(4) $$g\left(\alpha \right)\equiv \int \frac{d\alpha }{f\left(\alpha \right)}=\frac{A}{\beta }\int \exp\left(\frac{-E}{RT}\right)dT$$

Equation (4) expresses the famous temperature integral. Since there is no analytical solution for this integral, some approximate solution methods were derived in the era when computer numerical solution methods were underdeveloped [32].

The most popular kinetic analysis methods are model-free isoconversional methods in accordance with the isoconversional principle that the reaction rate at a given extent of conversion is only a function of temperature. Additionally, isoconversional methods have two main categories, including differential and integral. The differential methods take the natural logarithm through Equation (3) and yield Equation (5), which is the most commonly used differential isoconversional method, also known as Friedman [40]. At any given α, $E_{\alpha }$ can be obtained by the slope of $\ln{\left(\beta d\alpha /dT\right)}_{\alpha ,i}$ and $1/T_{\alpha ,i}$. The index i is introduced to denote different temperature programs.

(5) $$\ln\left(\frac{d\alpha }{dt}\right)=\ln{\left(\beta \frac{d\alpha }{dT}\right)}_{\alpha ,i}=\ln\left(A_{\alpha }f\left(\alpha \right)\right)-\frac{E_{\alpha }}{RT_{\alpha ,i}}$$

The integral isoconversional methods originate from the application of the isoconversional principle to Equation (4). From approximations of the temperature integral, the general form of Equation (4) after taking the natural logarithm can be written as Equation (6):

(6) $$\ln\left(\frac{\beta_{i}}{T^{B}{}_{\alpha ,i}}\right)=Const-C\left(\frac{E_{\alpha }}{RT_{\alpha ,i}}\right)$$

Different approximation methods lead to different equation forms like Flynn–Wall–Ozawa (FWO) [41,42,43], Kissinger–Akahira–Sunose (KAS) [44] and Starink [45], as summarized in Table 2. At any given α, $E_{\alpha }$ can be determined according to the slope of $\ln\left(\beta_{i}/T^{B}{}_{\alpha ,i}\right)$ and $1/T_{\alpha ,i}$ using all of the heating rate data in accordance with the isoconversional principle, and the index i is introduced to express different temperature programs.

Although the model-free method can obtain the activation energy without presupposing the reaction model, it is useless to determine the reaction model. The master plots method is a convenient way to confirm the reaction model [46,47]. Master plots are referenced theoretical curves that are only dependent on the reaction model, whereas they are independent of the pre-exponential factor A and the activation energy $E_{a}$. This method uses Equation (7) to transform the experimental data into experimental master plots and compare them with the theoretical curves to determine the reaction model.

(7) $$\frac{Z\left(\alpha \right)}{Z\left(0.5\right)}=\frac{f\left(\alpha \right)g\left(\alpha \right)}{f\left(0.5\right)g\left(0.5\right)}={\left(\frac{T_{\alpha }}{T_{0.5}}\right)}^{2}\frac{{\left(d\alpha /dt\right)}_{\alpha }}{{\left(d\alpha /dt\right)}_{0.5}}$$

where the subscript 0.5 represents the experimental data at α=0.5. The left-hand side of Equation (7) is the theoretical master plots, which represent the characteristics of the reaction model listed in Table 1. The right-hand side of Equation (7) is obtained with the experimental data. The most suitable reaction model can be determined through the comparison of the coefficient of determination ($R^{2}$) between the theoretical master plots and the experimental plots [48].

After the activation energy and reaction model are determined, the pre-exponential factor can be obtained using the Málek method [49,50]. In Equation (8), the subscript max denotes data related to the peak value of the differential thermogravimetric (DTG) or differential scanning calorimetry (DSC) curve at a given heating rate. Moreover, the pre-exponential factor can be directly obtained using Equation (3).

(8) $$A=-\frac{\beta E_{0}}{RT_{\max}^{2}f^{\prime }\left(\alpha_{\max}\right)}\exp\left(\frac{E_{0}}{RT_{\max}}\right)$$

### 2.2. Model of Pyrolysis Prediction

As mentioned before, kinetic analysis aims to establish the relationship between conversion rate and temperature by solving the kinetic triplet, while the most important practice application of pyrolysis kinetic analysis is kinetic prediction. Vyazovkin et al. [51] employed nonisothermal data to predict the epoxy curing time under isothermal conditions as Equation (9). Granado et al. [52] utilized isothermal data for nonisothermal kinetic predictions with the hypothesis that nonisothermal processes can be divided into a series infinitesimal step.

(9) $$t_{\alpha }=\frac{\frac{1}{\beta }\int_{0}^{T_{\alpha }}\exp\left(\frac{-E_{\alpha }}{RT}\right)dT}{\exp\left(\frac{-E_{\alpha }}{RT_{0}}\right)}$$

Since the temperature integral is suitable for the case of constant heating rates, the effective mathematical expression of conversion in an arbitrary temperature program has not been developed.

Dong [34] deduced the recursive mathematical relationship of pyrolysis degree based on the principle of Scheil sum as Equation (10) to calculate the real-time pyrolysis of composites during thermal decomposition, while the isothermal assumption was used in solving the temperature integral, as shown in Equation (11). Moreover, a virtual time as Equation (12) was introduced in this article, and its physical meaning or mathematical rationality were not clear. It violates the premise of non-constant temperature rise of composites during a lightning strike, and introduces errors into the calculation of pyrolysis degree.

(10) $$C_{i}=\left\{\begin{array}{cc} 1-{\left[(n-1)A\exp(-\frac{E_{a}}{RT_{1}})\Delta t+C_{0}\right]}^{\frac{1}{1-n}} & \left(i=1\right)\\ 1-{\left[{\left(1-C_{i-1}\right)}^{1-n}+(n-1)A\exp(-\frac{E_{a}}{RT_{i}})\Delta t\right]}^{\frac{1}{1-n}} & \left(i>1\right) \end{array}\right.$$

(11) $$\int \frac{dC}{{\left(1-C\right)}^{n}}=\frac{{\left(1-C\right)}^{1-n}}{n-1}=\int A\exp\left(-\frac{E_{a}}{RT}\right)+C_{0}=A\exp\left(-\frac{E_{a}}{RT}\right)t+C_{0}$$

(12) $$t_{2}^{*}=\frac{{\left(1-C_{1}\right)}^{1-n}-C_{0}}{\left(n-1\right)A\exp\left(-\frac{E}{RT_{2}}\right)}$$

Kamiyama [38] derived the mathematical relationship of multi-step kinetic pyrolysis degree with time under isothermal conditions based on an n-th order reaction model to calculate the real-time pyrolysis of composites during a lightning strike, as shown in Equation (13); it is obvious that this model cannot deal with pyrolysis with a nonlinear heating rate, and this will also introduce significant errors.

(13) $$\begin{array}{l} C=1-\sum_{k=1}^{N}f_{k}{\left\{(n_{k}-1)A_{k}\exp(-\frac{E_{k}}{RT})t+1\right\}}^{\frac{1}{1-n_{k}}}\\ \sum f_{k}=1 \end{array}$$

To derive the expression of pyrolysis degree in an arbitrary temperature program, it is necessary to start from the general form of the rate equation, Equation (3), and in this case, the temperature T is a function of time t, and Equation (3) can be rearranged as follows:

(14) $$\frac{d\alpha }{dt}=A\;\exp\left(\frac{-E}{RT\left(t\right)}\right)f\left(\alpha \right)$$

Integration of Equation (14) leads to:

(15) $$g\left(\alpha \right)\equiv \int_{0}^{\alpha }\frac{d\alpha }{f\left(\alpha \right)}=A\int_{0}^{t}\exp\left(\frac{-E}{RT\left(t\right)}\right)dt+C$$

where C denotes a constant. Equation (15) can be solved directly by numerical integration methods. However, the solution process is very complex and difficult to implement in the actual analysis process. Obtaining a difference between $g\left(\alpha_{i}\right)$ and $g\left(\alpha_{i-1}\right)$ corresponding to $t_{i}$ and $t_{i-1}$ to deduce the recurrence formula yields Equation (16).

(16) $$g\left(\alpha_{i}\right)-g\left(\alpha_{i-1}\right)=A\left(\int_{0}^{t_{i}}\exp\left(-\frac{E}{RT\left(t\right)}\right)dt-\int_{0}^{t_{i-1}}\exp\left(-\frac{E}{RT\left(t\right)}\right)dt\right)=A\int_{t_{i-1}}^{t_{i}}\exp\left(-\frac{E}{RT\left(t\right)}\right)dt$$

Obtaining an approximation of Equation (16) based on the trapezoidal integral formula yields Equation (17):

(17) $$g\left(\alpha_{i}\right)-g\left(\alpha_{i-1}\right)=\frac{A}{2}\left(\exp\left(-\frac{E}{RT\left(t_{i-1}\right)}\right)+\exp\left(-\frac{E}{RT\left(t_{i}\right)}\right)\right)\Delta t$$

where $\Delta t=t_{i}-t_{i-1}$. The expression of $g\left(\alpha \right)$ is selected from Table 1, while different reaction models may have different expressions and solved algorithms. If the D2 or D4 reaction model listed in Table 1 is selected, $\alpha_{i}$ cannot be expressed explicitly as a function of temperature and time; then, an iterative solution is required. For the selection of the n-th (n ≠ 1) order reaction model, since it is the most common reaction model for solid pyrolysis, Equation (17) is rearranged as:

(18) $$\frac{{\left(1-\alpha_{i}\right)}^{1-n}}{n-1}-\frac{{\left(1-\alpha_{i-1}\right)}^{1-n}}{n-1}=\frac{A}{2}\left(\exp\left(-\frac{E}{RT\left(t_{i}\right)}\right)+\exp\left(-\frac{E}{RT\left(t_{i-1}\right)}\right)\right)\Delta t$$

Subsequently, the numerical computation expression for obtaining the conversion degree is derived as Equation (19):

(19) $$\alpha_{i}=\left\{\begin{array}{cc} 0 & \left(i=0,\;t_{0}=0\right)\\ 1-{\left(\frac{\left(n-1\right)}{2}A\left(\exp\left(-\frac{E}{RT\left(t_{i}\right)}\right)+\exp\left(-\frac{E}{RT\left(t_{i-1}\right)}\right)\right)\Delta t+{\left(1-\alpha_{i-1}\right)}^{1-n}\right)}^{\frac{1}{1-n}} & \left(i>0,\;t_{i}>0\right) \end{array}\right.$$

The proposed model of pyrolysis prediction uses a simple mathematical approximation in the derivation process, and there are no requirements for the initial temperature and constant heating rate as the classical temperature integrations required [32]. The assumption of the trapezoidal integration method indicates that $K\left(T\right)$, not the temperature T, is approximately linear within a given time interval, so this prediction model applies to an arbitrary temperature program. Equation (19) is another form of the numerical integration method, and it can be easily implemented in the heat transfer analysis subroutine of commercial finite element software, such as Abaqus and ANSYS.

It is worth noting that the obtained expressions of conversion degree will be distinct if different reaction models are selected. In general, different materials may require different reaction models to be selected, which is only dependent on the reaction. The calculation accuracy will not be affected by the reaction model; what really matters is the time interval. With the use of the trapezoidal integral formula, the time step should be small enough to reduce the cumulative error.

## 3. Experimental Section

### 3.1. Materials

Laminate specimens were made using unidirectional carbon/epoxy prepreg tape (USN15000/9A16/RC33) supplied by Weihai GuangWei Composites Co., Ltd. The specimens were developed with the use of the above prepreg with unidirectional lay-up patterns [0]_6._ After lay-up, the prepreg stacks were cured for 180 min at a temperature of 120 °C and 3 standard atmospheric pressures in the autoclave.

### 3.2. Experimental Procedures

The thermogravimetric experiment was conducted on a TG thermal analyzer (NETZSCH STA 449 F3). The temperature of the sample used for TG analysis ranges was increased from 308.15 K to 1523.15 K at constant heating rates of 10, 20, 30 and 40 K/min. To obtain pyrolysis data for carbon fiber-reinforced epoxy resin composites under nonlinear heating, the temperature of the sample with nonlinear heating was increased from 308.15 K to 1523.15 k, with a heating rate of 10 K/min between 308.15 K and 638.15 K, 20 K/min between 638.15 K and 708.15 K, and 40 K/min between 708.15 K and 1523.15 K. All the test was performed in a nitrogen atmosphere with a purge gas flow rate of 60 mL/min and a protective gas flow rate of 20 mL/min, which could ensure that the sample was surrounded by nitrogen.

## 4. Results and Discussion

### 4.1. Thermal Decomposition Data Analysis

**Figure 1:**
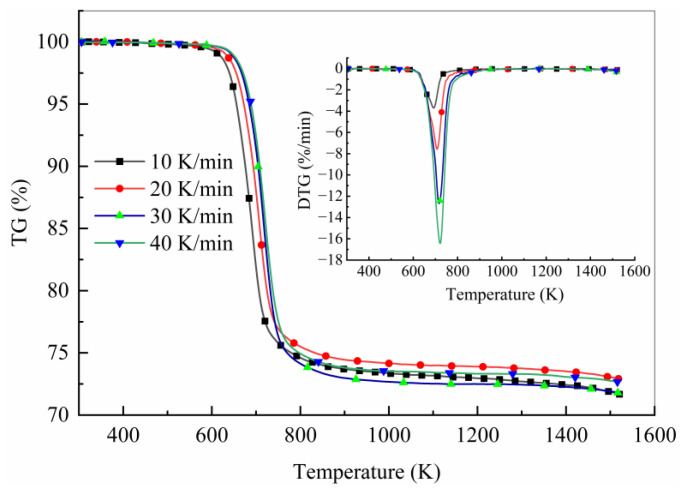
TG curves for USN15000/9A16/RC33 composite materials measured in N_2_ environment at various heating rates. Inset: corresponding DTG curves.

Figure 1 presents the thermogravimetric and differential thermogravimetric curves for the carbon fiber-reinforced epoxy composites at different constant rates of heating. Due to the uneven distribution of resin in the manufacturing process, the residual mass fraction was inconsistent at different heating rates. Since the pyrolysis degree was calculated by normalization, it did not affect the subsequent analysis. The carbon fiber-reinforced epoxy composites began to degrade at about 500 K, and the peak value of the weight loss rate at the respective heating rate appeared in the range of 650 K to 750 K. Moreover, the peak weight loss rate shifted to a higher temperature, and the mass loss rate increased with the increase in the heating rate. This above shift was primarily due to the time and temperature history that the materials were subjected to [48]. The lower the heating rate, the longer the time required to reach the same temperature.

**Figure 2:**
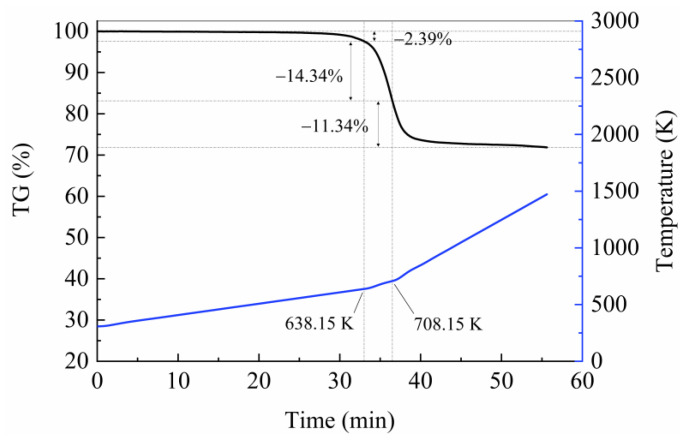
TG and temperature curves for USN15000/9A16/RC33 composite materials measured in N2 environment at the combined heating rates.

Figure 2 shows the thermogravimetric (TG) and temperature curves for the carbon fiber-reinforced epoxy resin composites at combined heating rates of 10, 20, and 40 K/min. Most of the pyrolysis process occurred at a heating rate of 20 K/min, and the temperature range of this process was very narrow. This undoubtedly increased the difficulty of kinetics prediction. It is worth noting that although the preset heating rates varied at 638.15 K and 708.15 K, there was a lag in the actual transition temperature due to the time required for the testing equipment to adapt to changes in the temperature program.

### 4.2. Determination of Kinetic Triplet

**Figure 3:**
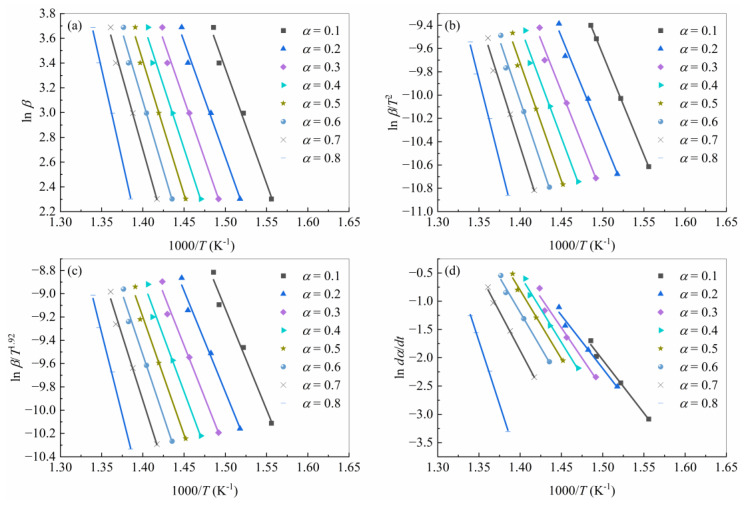
Isoconversional curves adopted for calculation of the activation energy with the (**a**) FWO method, (**b**) KAS method, (**c**) Starink method, and (**d**) Friedman method.

Based on isoconversional methods (FWO/KAS/Starink/Friedman) to obtain the activation energy, the extent of conversion was selected in the range of 0.1 to 0.8. The linear plots of $\ln\left(\beta_{i}/T_{\alpha ,i}^{B}\right)$ versus $1/T_{\alpha ,i}$ and $\ln{\left(d\alpha /dt\right)}_{\alpha ,i}$ versus $1/T_{\alpha ,i}$ are presented in Figure 3, respectively.

**Figure 4:**
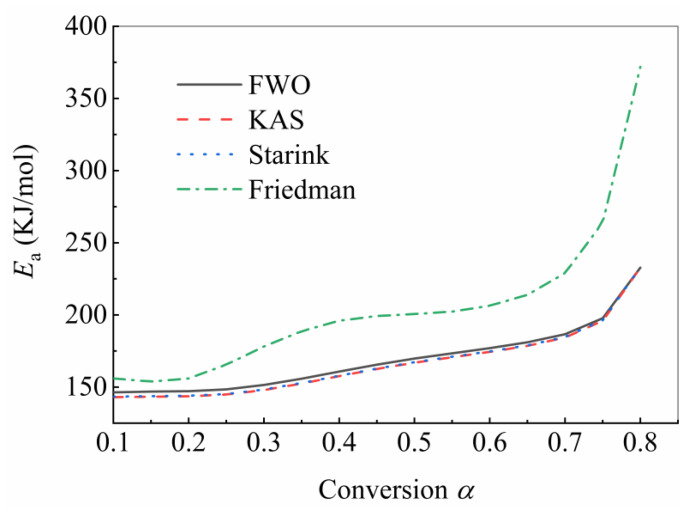
Evolution of the activation energy of USN15000/9A16/RC33 thermal decomposition using various isoconversional approaches.

For the above approach, $E_{\alpha }$ was calculated from the slope of the curves generated by Equations (5) and (6). Figure 4 illustrates the evolution of the activation energies calculated using isoconversional methods (FWO/KAS/Starink/Friedman) as a function of the extent of conversion α. The activation energy results obtained by the three integral methods (FWO/KAS/Starink) were basically consistent, while the activation energy predicted by Freedman was higher than those of the other three methods. For the FWO method, the activation energy increased from 146.4 kJ/mol to 232.7 kJ/mol with the extent of conversion from 0.1 to 0.8. The average activation energy was 164.9 kJ/mol. The ICTAC Kinetics Committee [23] recommends that an unremarkable change of $E_{\alpha }$ with α suggests that the process can be treated as a single-step reaction during the calculation of dynamic parameters. As listed in Figure 4, the activation energy increased slightly with the increase in the extent of conversion between 0.1 and 0.75, and the activation energy increased rapidly after the extent of conversion exceeding 0.75, which suggests that the thermal decomposition of USN15000/9A16/RC33 may be a multiple-step reaction. However, due to the predominance of the single-step process, the reaction model was determined based on the assumption that the reaction is single-step [53].

**Figure 5:**
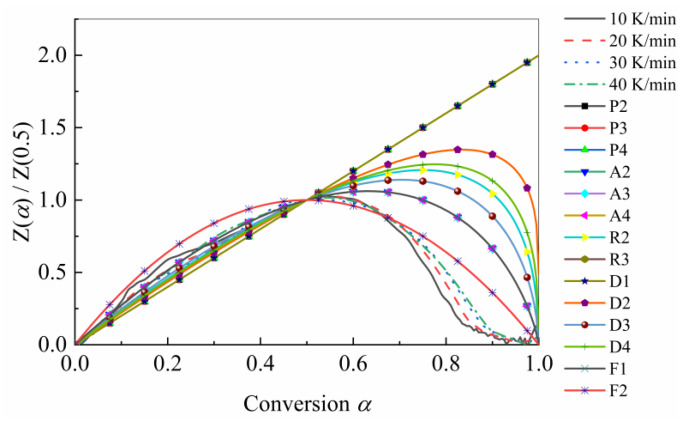
Master plots of different reaction models and experimental data in terms of USN15000/9A16/RC33 thermal decomposition.

**Figure 6:**
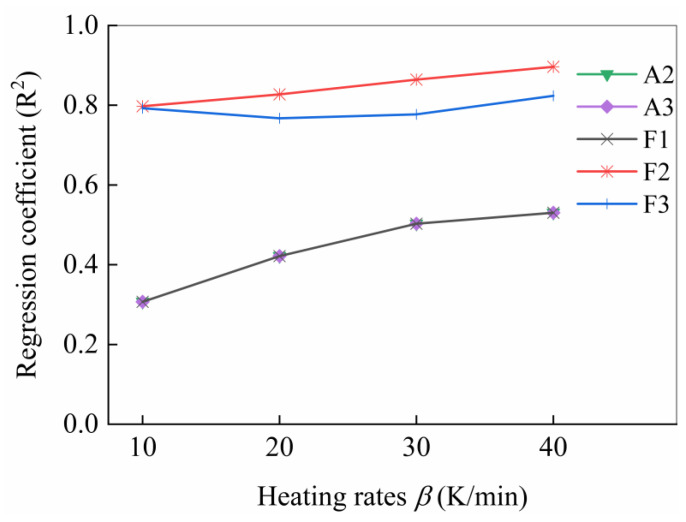
Regression coefficient (*R*^2^) between experimental data and master plots of various reaction models. The reaction model with regression coefficient less than zero was ignored.

To determine the reaction model with master plots, all of the reaction models used in the master plots method were selected from Table 1. Figure 5 presents the theoretical master plot $Z\left(\alpha \right)/Z\left(0.5\right)$ versus α for a variety of reaction models and experiment plots in terms of all four heating rates. The choice of the final reaction model followed the regression coefficient ($R^{2}$) between experimental and theoretical master plots based on all of the heating rates, as shown in Figure 6. As depicted in the figure, the second-order (F2) reaction model had the optimal fit goodness, so the F2 reaction model was selected as the reaction model for USN15000/9A16/RC33.

Once the activation energy and reaction model had been obtained, the pre-exponential factor could be obtained by substituting the activation energy into Equations (3) or (8). The value of the pre-exponential factor calculated by these two approaches is listed in Table 3. The range of pre-exponential factors predicted by these two approaches was roughly the same. However, when Equation (3) was used, the test data were adopted to obtain the pre-exponential factor without any approximation or simplification. Thus, it is suggested that the pre-exponential factor obtained by Equation (3) is more authentic. The pre-exponential factor obtained by the activation energy of the Friedman method was at least one order of magnitude larger than that of other methods.

**Figure 7:**
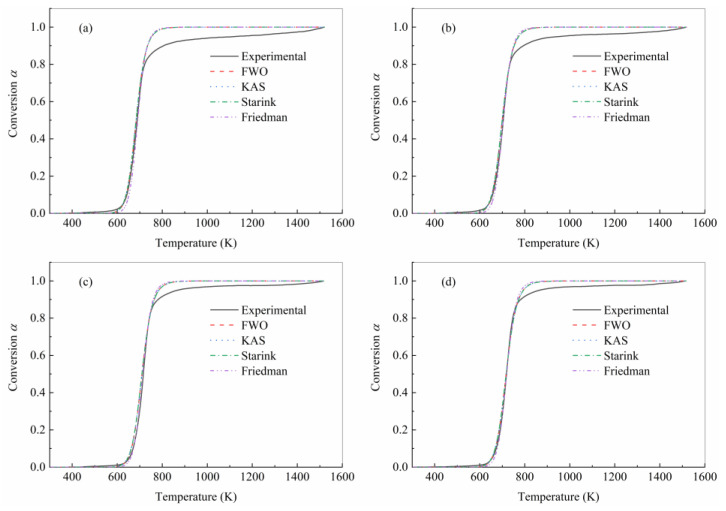
Comparison between experimental and theoretical curves under different heating rates by (**a**) 10 K/min, (**b**) 20 K/min, (**c**) 30 K/min, and (**d**) 40 K/min.

To verify and compare the accuracy of isoconversional methods, it is necessary to substitute the three obtained kinetic parameters into the equation, and then the numerical integration method should be adopted to generate the theoretical conversion curve. However, the data listed in Table 3 indicated that the values of the triple kinetic parameters were dispersed with the change in conversion; in particular, the pre-exponential factors under different conversion rates differed by several orders of magnitude. It is not appropriate to simply average the obtained values of the triple kinetic parameters to determine the final value. Considering that the pyrolysis was more stable when α=0.5, the triple kinetic parameters obtained at different heating rates were averaged, and the parameters when α=0.5 was taken as the reference value were as listed in Table 4. Figure 7 compares the theorical results with experimental data for all four heating rates. The theoretical curves generated using the four isoconversional methods were closely consistent with the experimental curves at the pyrolysis degree lower than 0.8. After the pyrolysis degree exceeding 0.8, there was a deviation between the theoretical curves and the experimental curves. The results were consistent with previous research [7,9]. The possible reason for this result is that the reaction mechanism is complex at the end of the reaction, and there was a certain error when the second-order model was adopted to approximate the reaction mechanism. However, using multi-step kinetics for analysis in pursuit of data consistency may not necessarily lead to a comprehensive understanding of the reaction mechanism, but rather lead to over-optimization of the data. Although there was a certain error between the theoretical and experimental results obtained, it still provides new reference for many engineering studies that lack effective characterization parameters, and this error is also acceptable in these fields.

### 4.3. Validation of Pyrolysis Prediction Model

**Figure 8:**
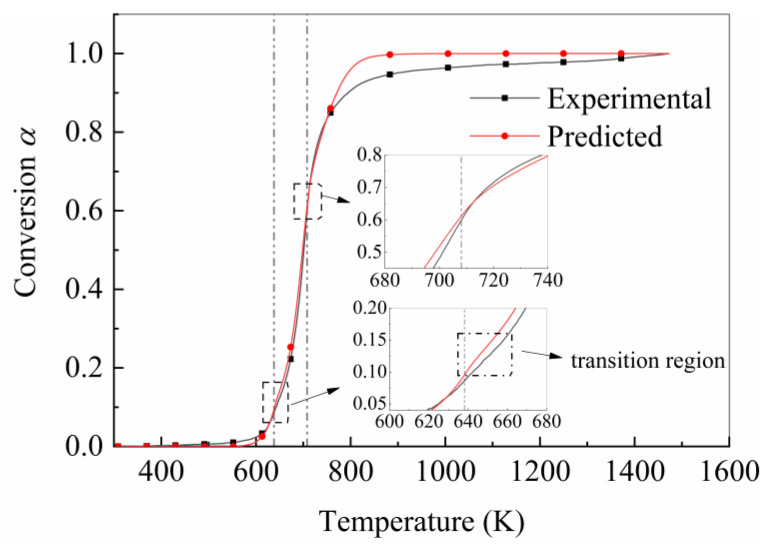
Comparison between experimental and theoretical curves under combined heating rates of 10, 20, and 40 K/min. The dashed line represents the temperature at which the heating rate undergoes a transition.4.4. Influence of Time Step on Prediction Accuracy.

Figure 8 shows the comparison between the experimental and predicted curves under combined heating rates of 10, 20, and 40 K/min. The predicted curve and experimental curve had good consistency when the pyrolysis degree was below 0.8. When the temperature point was near 638.15 K, the prediction model effectively captured the shifting trend of the pyrolysis curve towards the high-temperature zone. The error between the experimental and predicted curves near 638.15 K was not caused by the prediction model, but by the theoretical and experimental results. When the temperature point was near 708.15 K, the pyrolysis curve was not significantly affected by the change in heating rate, and it is not possible to directly extract the consistency characteristics between the predicted results and experimental results from the graph.

### 4.4. Influence of Time Step on Prediction Accuracy

**Figure 9:**
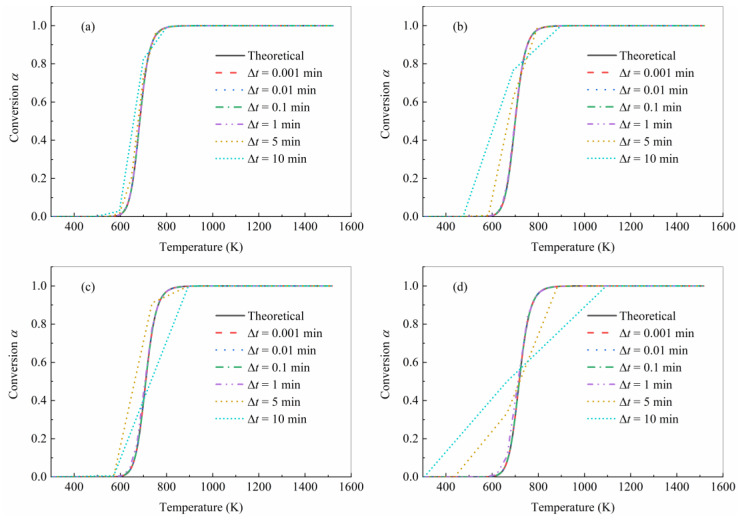
Comparison between theoretical and prediction curves under different heating rates by (**a**) 10 K/min, (**b**) 20 K/min, (**c**) 30 K/min, and (**d**) 40 K/min.

As previously stated, the model represented by Equation (19) can be applied to the calculation of pyrolysis degree in the heat transfer analysis subroutine of commercial finite element software. In the process of heat transfer finite element analysis, the time step and corresponding temperature rise will have a great impact on the analysis results, which will also affect the prediction accuracy of the pyrolysis degree. To verify the effect of time step and temperature rise on prediction accuracy, the theoretical curve obtained by the FWO method was used as the reference to compare the consistency of the prediction curve with the theoretical curve at different time intervals, as shown in Figure 9. Since the sampling time interval for the original data was 0.005 min, six sampling time intervals of 0.001 min, 0.01 min, 0.1 min, 1 min, 5 min, and 10 min were adopted for the predicted model. It should be noted that the temperature of the sampling point was obtained by interpolation in the real temperature program. Therefore, there may be a deviation between the actual value and the theoretical value of the temperature.

The predicted curves under lower time intervals (Δt=0.001 min, 0.01 min, 0.1 min, 1 min ) for all heating rates were closely consistent with the theoretical curves. However, at the larger sampling intervals (Δt= 5 min, 10 min), the error in the high temperature heating rates increased significantly with the increase in the temperature change in the time intervals. For Δt=10 min at the heating rate of 10 K/min, when the temperature increased from 596.2 K to 699.5 K between two sampling points, most of the pyrolysis was completed in this temperature range; with the conversion degree changed from 0.0286 to 0.8243, the shape error between the predicted curve and the theoretical curve was still acceptable. Nevertheless, for Δt=10 min at the heating rate of 40 K/min, the conversion degree changed from 0.000 to 0.500 with the temperature increase from 308.15 K to 664.67 K between two sampling points, and the coincidence between the prediction curve and the simulation curve was low. It is worth noting that under all four heating rates, the temperature range of the main pyrolysis process was about 600 K to 800 K, and compared with the whole sample heating process, the temperature range of pyrolysis was narrow. Therefore, capturing the starting point and end point of the main pyrolysis process is very critical to the prediction accuracy. For Δt=10 min at the heating rate of 10 K/min, the time interval can effectively capture the temperature at the beginning and end of the main pyrolysis process, while it cannot do so for Δt=10 min at the heating rate of 40 K/min. In brief, a reasonable time interval should be employed to ensure that the temperature change is not too dramatic during the sampling interval, and the temperature of the main pyrolysis process starting and ending points will be captured.

## 5. Conclusions

In this study, the thermal decomposition behavior of carbon fiber-reinforced epoxy composites at heating rates of 10 K/min to 40 K/min was investigated, and a model of pyrolysis prediction suitable for arbitrary temperature programs was established.

Carbon fiber-reinforced epoxy composites began to degrade at nearly 500 K, and the peak value of the weight loss rate at the respective heating rate appeared form 650 K to 750 K. The lone peak of the DTG plot suggests that the overall decomposition was a single-step reaction, while the activation energy increased rapidly when the conversion exceeded 0.75, which indicates that the thermal decomposition of USN15000/9A16/RC33 may be a multiple-step reaction. Due to the predominance of the single-step process, it is still assumed that the reaction was a single-step reaction, and the theoretical results indicated that the error was within the acceptable range. Although there were differences in the kinetic parameters of carbon fiber-reinforced epoxy composites derived using different isoconversional methods (KAS/FWO/Starink/Friedman), all of them were capable of characterizing the thermal decomposition behavior of the material.

The proposed pyrolysis prediction model can avoid many numerical limitations of temperature integration and is suitable for arbitrary temperature programs. The effectiveness of the pyrolysis prediction model was verified through the results of thermogravimetric experiments with combined heating rates. By adopting an appropriate sampling time interval, the temperature change between sampling intervals was not violent, so the results of the prediction model were closely consistent with the theoretical results. The proposed pyrolysis prediction model is mainly for single-step reactions, and its applicability to multi-step reactions needs to be further verified.

## Figures and Tables

**Table 1:** Most commonly used reaction models in solid-state kinetics.

Reaction Model	Model Code	*f* (*α*)	*g* (*α*)
Power law	P2	$2\alpha^{1/2}$	$\alpha^{1/2}$
Power law	P3	$3\alpha^{2/3}$	$\alpha^{1/3}$
Power law	P4	$4\alpha^{3/4}$	$\alpha^{1/4}$
Avrami–Erofeyev	A2	$2\left(1-\alpha \right){\left[-\ln\left(1-\alpha \right)\right]}^{1/2}$	${\left[-\ln\left(1-\alpha \right)\right]}^{1/2}$
Avrami–Erofeyev	A3	$3\left(1-\alpha \right){\left[-\ln\left(1-\alpha \right)\right]}^{2/3}$	${\left[-\ln\left(1-\alpha \right)\right]}^{1/3}$
Avrami–Erofeyev	A4	$4\left(1-\alpha \right){\left[-\ln\left(1-\alpha \right)\right]}^{3/4}$	${\left[-\ln\left(1-\alpha \right)\right]}^{1/4}$
Contracting cylinder	R2	$2{\left(1-\alpha \right)}^{1/2}$	$1-{\left(1-\alpha \right)}^{1/2}$
Contracting sphere	R3	$3{\left(1-\alpha \right)}^{2/3}$	$1-{\left(1-\alpha \right)}^{1/3}$
One-dimensional diffusion	D1	$1/2\alpha^{-1}$	$\alpha^{2}$
Two-dimensional diffusion	D2	${\left[-\ln\left(1-\alpha \right)\right]}^{-1}$	$\left(1-\alpha \right)\ln\left(1-\alpha \right)+\alpha$
Three-dimensional diffusion	D3	$3/2{\left(1-\alpha \right)}^{2/3}{\left[1-{\left(1-\alpha \right)}^{1/3}\right]}^{-1}$	${\left[1-{\left(1-\alpha \right)}^{1/3}\right]}^{2}$
Ginstling–Brounshtein	D4	$3/2{\left[{\left(1-\alpha \right)}^{-1/3}-1\right]}^{-1}$	$1-\left(2\alpha /3\right)-{\left(1-\alpha \right)}^{2/3}$
First-order	F1	1−α	$-\ln\left(1-\alpha \right)$
Second-order	F2	${\left(1-\alpha \right)}^{2}$	${\left(1-\alpha \right)}^{-1}-1$
Third-order	F3	${\left(1-\alpha \right)}^{3}$	$[{\left(1-\alpha \right)}^{-2}-1]/2$

**Table 2:** List of isoconversional models used in this study.

		Method	Expression
Isoconversional methods	Differential	Friedman	$\ln{\left(\frac{d\alpha }{dt}\right)}_{\alpha ,i}=\ln{\left(\beta \frac{d\alpha }{dT}\right)}_{\alpha ,i}=\ln\left[A_{\alpha }f\left(\alpha \right)\right]-\frac{E_{\alpha }}{RT_{\alpha ,i}}$
	Integral	Flynn–Wall–Ozawa (FWO)	$\ln\left(\beta_{i}\right)=\;\mathrm{Const}\;-1.052\left(\frac{E_{\alpha }}{RT_{\alpha ,i}}\right)$
		Kissinger–Akahira–Sunose (KAS)	$\ln\left(\frac{\beta_{i}}{T_{\alpha ,i}^{2}}\right)=\;\mathrm{Const}\;-\frac{E_{\alpha }}{RT_{\alpha ,i}}$
		Starink	$\ln\left(\frac{\beta_{i}}{T_{\alpha ,i}^{1.92}}\right)=\;\mathrm{Const}-1.0008\left(\frac{E_{\alpha }}{RT_{\alpha ,i}}\right)$

**Table 3:** Kinetic parameters obtained using various isoconversional approaches.

Heating Rate	Isoconversional Method	$E_{\alpha }$ (kJ/mol)	$A_{\alpha }$ (min^−1^)		*f* (*α*)
			Equation (3) (Differential)	Equation (8) (Málek)	
10 K/min	FWO	146.4–232.7	$4.5\times 10^{10}$ $–6.4\;\times 10^{16}$	$4.6\times 10^{10}$ $–2.4\;\times 10^{17}$	${\left(1-\alpha \right)}^{2}$
	KAS	143.1–232.6	$2.4\times 10^{10}$ $–6.3\;\times 10^{16}$	$2.5\times 10^{10}$ $–2.4\;\times 10^{17}$	
	Starink	143.4–232.9	$2.6\times 10^{10}$ $–6.7\;\times 10^{16}$	$2.7\times 10^{10}$ $–2.5\;\times 10^{17}$	
	Friedman	156.0–371.9	$2.7\times 10^{11}$ $–7.7\;\times 10^{26}$	$2.6\times 10^{11}$ $–1.3\;\times 10^{28}$	
20 K/min	FWO	146.4–232.7	$4.7\times 10^{10}$ $–1.0\;\times 10^{17}$	$5.0\times 10^{10}$ $–1.9\;\times 10^{17}$	
	KAS	143.1–232.6	$2.6\times 10^{10}$ $–1.0\;\times 10^{17}$	$2.8\times 10^{10}$ $–1.9\;\times 10^{17}$	
	Starink	143.4–232.9	$2.7\times 10^{10}$ $–1.0\;\times 10^{17}$	$2.9\times 10^{10}$ $–2.0\;\times 10^{17}$	
	Friedman	156.0–371.9	$2.7\times 10^{11}$ $–8.4\;\times 10^{26}$	$2.7\times 10^{11}$ $–5.8\;\times 10^{27}$	
30 K/min	FWO	146.4–232.7	$4.5\times 10^{10}$ $–1.3\;\times 10^{17}$	$5.0\times 10^{10}$ $–1.6\;\times 10^{17}$	
	KAS	143.1–232.6	$2.5\times 10^{10}$ $–1.2\;\times 10^{17}$	$2.8\times 10^{10}$ $–1.5\;\times 10^{17}$	
	Starink	143.4–232.9	$2.6\times 10^{10}$ $–1.3\;\times 10^{17}$	$2.9\times 10^{10}$ $–1.6\;\times 10^{17}$	
	Friedman	156.0–371.9	$2.5\times 10^{11}$ $–7.8\;\times 10^{26}$	$2.7\times 10^{11}$ $–3.6\;\times 10^{27}$	
40 K/min	FWO	146.4–232.7	$5.2\times 10^{10}$ $–1.4\;\times 10^{17}$	$5.8\times 10^{10}$ $–1.7\;\times 10^{17}$	
	KAS	143.1–232.6	$2.9\;\times 10^{10}$ $–1.4\;\times 10^{17}$	$3.3\times 10^{10}$ $–1.6\;\times 10^{17}$	
	Starink	143.4–232.9	$3.1\;\times 10^{10}$ $–1.4\;\times 10^{17}$	$3.5\times 10^{10}$ $–1.7\;\times 10^{17}$	
	Friedman	156.0–371.9	$2.9\;\times 10^{11}$ $–7.7\;\times 10^{26}$	$3.1\times 10^{11}$ $–3.3\;\times 10^{26}$	

**Table 4:** Kinetic parameters used to verify and compare the accuracy of isoconversional methods.

Isoconversional Method	$E_{\alpha }$ (kJ/mol)	$A_{\alpha }$ (min^−1^)	*f* (*α*)
	α=0.5	Equation (3) (Differential)	
FWO	169.80	$4.5\;\times 10^{12}$	${\left(1-\alpha \right)}^{2}$
KAS	166.94	$2.76\;\times 10^{12}$	
Starink	167.27	$2.93\;\times 10^{12}$	
Friedman	200.62	$8.21\;\times 10^{14}$	

## Data Availability

The data that support the findings of this study are available from the corresponding author upon reasonable request.
